# “Herbal seizures” – atypical symptoms after ibogaine intoxication: a case report

**DOI:** 10.1186/s13256-015-0731-4

**Published:** 2015-10-31

**Authors:** Lorenz Breuer, Burkhard S. Kasper, Bernd Schwarze, Juergen M. Gschossmann, Johannes Kornhuber, Helge H. Müller

**Affiliations:** 1 Medical Campus University of OldenburgSchool of Medicine and Health Sciences Psychiatry and Psychotherapy, University Hospital Karl-Jaspers-Klinik , Hermann-Ehlers-Strasse 7, Bad Zwischenahn, D-26160 Germany; 2 Friedrich-Alexander University of Erlangen-Nuremberg, Erlangen, Germany; 3 Department of Neurology, Friedrich-Alexander University Erlangen-Nuremberg, University Hospital Erlangen, Erlangen, Germany; 4 Department of Psychiatry and Psychotherapy, Friedrich-Alexander University Erlangen-Nuremberg, University Hospital Erlangen, Erlangen, Germany; 5 Epilepsy Center, Department of Neurology, Friedrich-Alexander University Erlangen-Nuremberg, University Hospital Erlangen, Erlangen, Germany; 6 Department of Forensic Medicine, Friedrich-Alexander University Erlangen-Nuremberg, Erlangen, Germany; 7 Department of Internal Medicine, Klinikum Forchheim/Friedrich-Alexander Universität Erlangen-Nuremberg, Erlangen, Germany

**Keywords:** Ibogaine misuse, Intoxication, Reactive seizures, Side effects

## Abstract

**Introduction:**

Misuse of various new psychotropic substances such as ibogaine is increasing rapidly. Knowledge of their negative side effects is sparse.

**Case presentation:**

We present a case of intoxication with the herbal substance ibogaine in a 22-year-old white man. After taking a cumulative dose of 38 g (taken in two doses), he developed visual memories, nausea and vomiting. He developed a generalized tonic–clonic seizure with additional grand mal seizures. He was treated with midazolam and levetiracetam. Extended drug screenings and computed tomography and magnetic resonance imaging findings were all negative.

**Conclusions:**

Knowledge of the side effects of ibogaine has mainly come from reports of cardiovascular complications; seizures are rarely mentioned and experimental findings are inconsistent. It seems that ibogaine acts like a proconvulsive drug at high doses.

## Introduction

The use of synthetic legal or semi-legal substances is increasing rapidly, especially in patients under the age of 30 years [1–4]. One such substance is ibogaine, a natural alkaloid extracted from the roots of the rain forest shrub *Tabernanthe iboga*. Ibogaine is known in alternative and rural medicine. In Gabon it is used for initiation ceremonies to induce a near-death experience for psychological purposes and to produce a rural-spiritual contact with the ancestors. It acts as a traditional “high” that includes hallucinations and feelings of depersonalization.

In Western countries, the substance is used off-label and experimentally for the specific indication of detoxification from opiates, stimulants, alcohol and nicotine; in particular, it is used to treat withdrawal symptoms [5, 6]. Anticonvulsive and stimulant *in vitro* and *in vivo effects* were described for ibogaine [7]. The most commonly used form is the hydrochloride salt of ibogaine (HCl), but alkaloid extracts or dried root bark are also used. Experimental findings suggested that ibogaine elevates plasma prolactin and corticosterone levels and that it is involved in decreasing dopamine (DA) neurotransmission. Ibogaine also decreases neurotransmission of serotonin receptors (5-hydroxytryptamine; 5-HT) in the striatum [7–11]. The anticonvulsive mode of action occurs via an N-methyl-d-aspartate (NMDA) receptor antagonism, a finding that has been well documented in experimental and therapeutic examinations [8, 12, 13].

The psychoactive state associated with ibogaine has been likened to a waking dream/dreamy state that sometimes includes interrogatory verbal exchanges. Another described experience is panoramic memory or the recall of rapid dense successions of autobiographical visual memories. These experiences have been associated with functional muscarinic cholinergic effects, which are prominent in the mechanisms of dreaming and memory [14, 15]. Ibogaine is used most frequently as a single oral dose in the range of 10 to 25 mg/kg of body weight. In the USA and most European countries, ibogaine is classified as an illegal drug [15, 16].

## Case presentation

We report the case of a 22-year-old white man in good physical health (body height of 184 cm; body weight of 76.6 kg) who used ibogaine for the first time; he had no history of acute or chronic illness and no history of any drug dependence. He had no concomitant use of prescribed medications. Prior to admittance he had not taken any somatic medication for approximately 6 months.

**Fig. 1:**
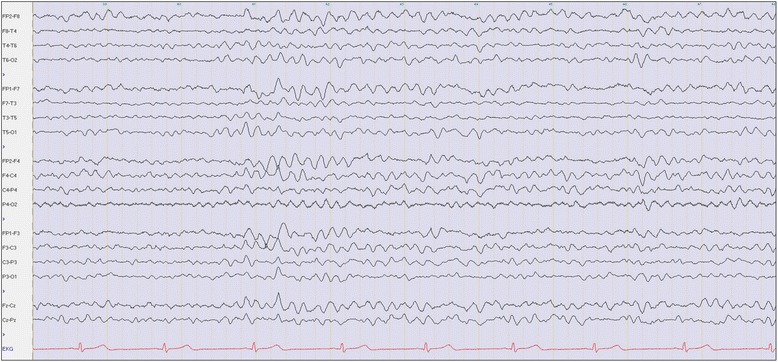
Electroencephalogram at day four after ibogaine intoxication. The electroencephalogram shows an irregular alpha rhythm and a significant portion of diffuse theta waves consistent with recent intoxication. No focal slowing and no epileptiform discharges are shown

According to his own statement, he wanted to achieve a “spiritual cleansing and reboot” by taking ibogaine. He had ordered dried ibogaine root bark via the Internet and took a cumulative dose of 38 g. He took two portions with <5 minute of latency between the two doses, grinding it and dissolving it in water. He reported having visions that occurred approximately 1 hour after he consumed the substance, which is a typical finding in ibogaine use [10, 11]. These visions, which mainly consisted of visual memories of his life, lasted for 10 minutes. Subsequently, he felt nauseated and had to vomit repeatedly. Over the next 30 minutes, the vomiting continued and he started to develop muscle tension and cramps in his arms and legs. Ten hours after he took the drug, his relatives found him having a generalized (tonic–clonic) seizure (please see also Fig. 1). The emergency physician administered midazolam intravenously. The patient was immediately admitted to our intensive care unit, where he had several grand mal seizures. Within the next 3 days, repeated administrations of midazolam were necessary to stop the persistent generalized seizures. Anticonvulsive treatment with levetiracetam (1000 mg) was initiated on day two and immediately stopped the symptoms He was constantly awake and had no further neurologic progression (for example tremor, clonus or hyperreflexia). Cranial computed tomography and magnetic resonance tomography did not show any pathological findings. All laboratory tests showed unspecific alterations which might be related to the multiple previous epileptic seizures. In addition, slightly increased C-reactive protein (CRP) values and white blood cell counts (including lymphocyte count and neutrophil count), mild decreased platelet counts and elevated creatine-kinase values were found temporarily during the first days after admission. No signs of an infection and no inflammatory process were found.

His neurological status on day two revealed mild dysarthria with a subjective feeling of heaviness of the tongue, mild bilateral ptosis and psychomotor slowness. An electroencephalogram (EEG) showed mild diffuse encephalopathic changes but no epileptiform activity. On day five, all symptoms had disappeared completely and the laboratory values had normalized. Only the already declining creatine-kinase remained slightly increased, which was consistent with his several previous tonic–clonic epileptic seizures. He was finally discharged from our hospital. During a 3-month control period after this event that included documented abstinence, he had no further seizures without levetiracetam treatment, which had been tapered off after a few weeks. Detailed information about clinical and laboratory findings is provided in Table 1.

**Table 1:** Apparatus and laboratory findings during the observation period after ibogaine intoxication

Type of analysis	Day 1	Day 2	Day 3	Day 4	Day 5	Month 3
Computed tomography	–	No pathological findings	–	–	–	
Magnetic resonance imaging	–	–	No pathological findings	–	–	
Electrocardiogram	Sinus tachycardia (>120 beats per minute)/Sinus rhythm	Sinus rhythm	Sinus rhythm 55 beats per minute; QTc: 100 %	–	–	
Electroencephalogram		–	–	Irregular alpha rhythm, significant portion of diffuse theta waves, no focal slowing, no epileptiform discharges	–	Regular alpha rhythm, no focal slowing, no epileptiform discharges
Laboratory findings						Reference values
White blood cell count (×10^3^/μl)	**11.7**	**10.6**	6.2	5.1	4.4	4.0–9.4
Platelet count (×10^3^/μl)	248	164	**136**	**127**	176	150–440
Lymphocyte count (%)	**5.8**	**11.7**	25	29.4	28.0	25–40
Neutrophil count (%)	**87.8**	**79.2**	62.4	59.9	58.8	50–75
Creatinine (mg/dl)	**1.24**	0.84	0.75	0.79	0.76	<1.2
Creatine-kinase (U/l)	–	–	**370**	**234**	**194**	<190
C-reactive protein (mg/l)	**0.69**	–	**0.71**	0.39	0.35	0.0–0.5
Carbohydrate-deficient transferrin (%)	–	–	–	–	1.59	<2.6
Ibogaine concentration in hair sample (pg/mg)			**22**			–
Noribogaine concentration in hair sample (pg/mg)			**70**			–
Noribogaine concentration in urine sample (ng/mg)			**9.2**			–

Pathological findings are marked in bold. All other routine parameters (for example liver enzymes, electrolytes, coagulation status, triglycerides, cholesterol, thyroid-stimulating hormone, free triiodothyronine, and free thyroxine), as well as the screening for drugs in the patient’s urine (benzodiazepine, amphetamine, morphine/opiate/heroin, barbiturates, ecstasy/3,4-methylenedioxy-methamphetamine, methadone, cocaine metabolites, methamphetamine, tetrahydrocannabinol, fentanyl, tricyclic antidepressants, buprenorphine), and in the serum (barbiturates, benzodiazepine, tricyclic antidepressants) showed no pathological findings

## Discussion

Knowledge about the potential side effects of ibogaine is sparse. Sudden deaths have been related to the use of ibogaine, mainly because of concomitant medication use and comorbidities (especially cardiovascular) and/or its use among drug users as a self-treatment for detoxification. Most reports have noted fatal cardiac symptoms with QT prolongation and cardiac arrhythmias [17, 18]. By contrast, our patient survived without any detected severe electrocardiogram (ECG) abnormalities. Alper *et al*. reported the case of a patient who presented observed and well-documented seizures directly related to ibogaine, which the patient did not take for detoxification purposes [19]. In addition to the known psychotropic effects, our patient showed additional reactive generalized epileptic seizures caused by ibogaine. He had no additional history of drug abuse, so a substance-induced trigger could be excluded. Also an interaction of ibogaine with prescribed substances as a potentiator of the seizure events could be excluded in this case.

This description of a seizure induction via ibogaine misuse is rare and is in contrast to the supposed anticonvulsive mode of action via NMDA receptor antagonism of ibogaine which seems to mediate the anticonvulsive effects of the substance [20, 21]. In clinical settings a paradoxical seizure exacerbation by anti-epileptic medication is a known clinical phenomenon but the cellular mechanisms still remain unclear [22, 23]. One possible explanation might be an enhanced disinhibition process by a dose-dependent suppression of inhibitory interneurons. Ibogaine like MK-801 (dizocilpine) also stimulates a release of glucocorticoids that in turn increase susceptibility to seizures. The seizures observed in our patient could be related to the same glucocorticoid effect [8].

Binienda *et al*. described that MK-801 (dizocilpine) which also acts as an NMDA antagonist, paradoxically enhances electrographic seizures that preceded suppression of status epilepticus [24].

Although we cannot provide the exact ibogaine concentration of the dried root bark that our patient used, a dose of 38 g is high compared with the doses of 2 to 30 g of dried ibogaine root bark reported in literature [25]. Assuming an ibogaine concentration of 7 %, as previously reported [26], this would have corresponded to a single ingestion dosage of 2260 mg, or 35 mg/kg. It can be assumed that in our patient’s case, ibogaine acted like a high-dose-dependent proconvulsive drug while in lower doses being generally supposed to have an anticonvulsive mode of action.

This finding could stimulate further experimental studies to examine this hypothesis of a dose-dependent convulsive mode of action of ibogaine.

## Conclusions

It seems that in addition to its psychotropic and cardiac risk profile, ibogaine also has a substantial proconvulsive mode of action that is atypical given its NMDA antagonism, and this is potentially dose dependent. Of course, the significance of this finding is limited because only one documented case exists. Nevertheless, more case examinations and experimental findings could probably prove this hypothesis of a dose-dependent proconvulsive mode of action of ibogaine in higher consumed doses. These upcoming examinations should integrate and control for experimental findings that postulate that ibogaine has anticonvulsive effects [7]. Overall, taking into account the rising consumption of extraordinary herbal substances and the increasing number of reported side effects, physicians should be aware of this clinical manifestation of ibogaine use.

## Consent

Written informed consent was obtained from the patient for publication of this case report and any accompanying images. A copy of the written consent is available for review by the Editor-in-Chief of this journal.
